# *Arabidopsis thaliana Myb59* Gene Is Involved in the Response to *Heterodera schachtii* Infestation, and Its Overexpression Disturbs Regular Development of Nematode-Induced Syncytia

**DOI:** 10.3390/ijms22126450

**Published:** 2021-06-16

**Authors:** Anita Wiśniewska, Kamila Wojszko, Elżbieta Różańska, Klaudia Lenarczyk, Karol Kuczerski, Mirosław Sobczak

**Affiliations:** 1 Department of Plant Physiology, Institute of Biology, Warsaw University of Life Sciences (SGGW), Nowoursynowska 159, 02-776 Warsaw, Poland; wojszko.kamila@gmail.com (K.W.); kla.lenarczyk@gmail.com (K.L.); karolkuczerski@gmail.com (K.K.); 2 Department of Botany, Institute of Biology, Warsaw University of Life Sciences (SGGW), Nowoursynowska 159, 02-776 Warsaw, Poland; elzbieta_rozanska@sggw.edu.pl (E.R.); miroslaw_sobczak@sggw.edu.pl (M.S.)

**Keywords:** *Arabidopsis thaliana*, cyst nematode, *Heterodera schachtii*, plant defense, transcription factor, MYB

## Abstract

Transcription factors are proteins that directly bind to regulatory sequences of genes to modulate and adjust plants’ responses to different stimuli including biotic and abiotic stresses. Sedentary plant parasitic nematodes, such as beet cyst nematode, *Heterodera schachtii*, have developed molecular tools to reprogram plant cell metabolism via the sophisticated manipulation of genes expression, to allow root invasion and the induction of a sequence of structural and physiological changes in plant tissues, leading to the formation of permanent feeding sites composed of modified plant cells (commonly called a syncytium). Here, we report on the *AtMYB59* gene encoding putative MYB transcription factor that is downregulated in syncytia, as confirmed by RT-PCR and a promoter p*Myb59::GUS* activity assays. The constitutive overexpression of *AtMYB59* led to the reduction in *A. thaliana* susceptibility, as indicated by decreased numbers of developed females, and to the disturbed development of nematode-induced syncytia. In contrast, mutant lines with a silenced expression of *AtMYB59* were more susceptible to this parasite. The involvement of ABA in the modulation of *AtMYB59* gene transcription appears feasible by several ABA-responsive *cis* regulatory elements, which were identified in silico in the gene promoter sequence, and experimental assays showed the induction of *AtMYB59* transcription after ABA treatment. Based on these results, we suggest that *AtMYB59* plays an important role in the successful parasitism of *H. schachtii* on *A. thaliana* roots.

## 1. Introduction

Sedentary plant parasitic nematodes employ the reprogramming of plant cell metabolism through a sophisticated manipulation of gene expression to achieve favorable conditions for the induction and development of their permanent feeding sites, which facilitate their own development and reproduction. They can cause extensive yield losses in almost all economically important crops [1]. The cyst-forming nematodes belong to *Globodera* and *Heterodera* genera, and they include some of the most economically harmful plant parasites such as potato cyst nematodes (*Globodera pallida* and *G. rostochiensis*), beet cyst nematode (*Heterodera schachtii*), soybean cyst nematode (*H. glycines*), and cereal cyst nematodes (*H. avenae* and *H. filipjevi*) [1]. Classical agrotechnical methods of cyst nematodes control (crop rotation, resistant cultivars, fallow, trap crops, solarization, intercropping, chemical nematicides, and/or biological control agents) are not effective enough, even when used in combination. The cropping of resistant cultivars is a method that is relatively easy, highly effective and inexpensive. However, its effectiveness and wide implementation are severely limited due to the scarcity of nematode-resistance genes that are species- and even pathotype-specific. Additionally, resistance granted by some nematode-resistance genes can easily be overcome by nematode populations during the permanent or repeated cultivation of the same crop, thus leading to the emergence of virulent pathotypes [1]. The infective second-stage juveniles (J2s) emerging from the eggs hidden in a protective cyst invade the root and migrate across the epidermis and cortex toward the vascular cylinder, where they select a single initial syncytial cell that incorporates neighboring parenchymatic and meristematic cells by the formation of local cell wall dissolution, thus giving rise to the syncytium. During the migration and selection of the initial syncytial cell, as well as during the feeding from the syncytium, the nematodes release secretions that are produced in their subventral and dorsal glands and promote the formation and functioning of the syncytium [2,3,4]. The syncytium is the only source of nutrients for nematodes during their whole lifetime. Two to three weeks after infection, after three molts, the juvenile develops into either an adult immobile female or a mobile vermiform male. After fertilization, females start to produce eggs and then die, turning into a protective cyst filled with eggs.

Transcription factors are proteins directly binding to *cis* regulatory elements mostly located in gene promoters in order to modify and regulate gene expression. Through the specific regulation of gene expression, they can control plant responses to biotic and abiotic stresses or modulate developmental processes. Most transcription factors have divergent functions in plants and animals. Approximately 45% of *A. thaliana* transcription factors belong to protein families specific to plants [5]. The *A. thaliana* genome contains 27,655 genes encoding proteins, among which 1700 (6%) genes encode transcription factors, including 339 genes encoding transcription factors with the MYB domain [5,6,7]. The MYB domain contains one to three repeats of 50–53 amino acids residues and is responsible for binding to DNA in a sequence-specific manner to regulate the expression of target genes [5,6,8]. Each of the MYB repeats, within the MYB domain, forms a helix–turn–helix secondary structure. The MYB domain is usually located at the N-terminus of MYB proteins. The C-terminal region of MYB proteins is highly variable and functions as either an activation or a repression domain. Among plant MYB family proteins, those with two MYB repeats predominate and are called R2R3-MYB proteins [5,6,8].

Phytohormones are involved in many aspects of plant development and responses to a wide range of biotic and abiotic stresses. The defense response of different plant species upon plant pathogen infection is often modulated by stress phytohormones: jasmonic acid (JA), ethylene (ET), salicylic acid (SA) and abscisic acid (ABA) [9,10]. The role of SA and JA in the plant regulation of basal defense response (pattern-triggered immunity (PTI)) or *R*-gene-mediated defense response (effector triggered immunity (ETI)) has been well characterized in the case of bacteria and fungi. The defense against biotrophic leaf pathogens generally involves SA-dependent signaling, whereas inducible defense against leaf-chewing insects and necrotrophic microbes was mediated by JA-dependent signaling [9]. However, exceptions and more complex events also exist. Furthermore, SA and JA signals frequently interact either antagonistically or synergistically [11].

The knowledge of the basic molecular mechanisms of interactions between plant and nematode may provide tools to develop new types of plant resistance or tolerance to nematode infection. Functional analyses of genes with different expression profiles during pathogenesis and the discovery of a relationship between a gene product and the nematode ability to develop in host roots can be useful for the selection of genes with fundamental importance for nematode parasitism. For detailed analysis, we selected one of the MYB genes on the basis of a list of differentially expressed genes published by Szakasits et al. [12] in a report concerning analyses of the transcriptome of syncytia induced by the beet cyst nematode *H. schachtii* in *A. thaliana* roots. Additionally, *AtMyb59* was shown to be downregulated during *A. thaliana* root infestation by root-knot nematode *Meloidogyne javanica*, which induces the development of giant cells [13]. The selected gene, *AtMYB59*, is downregulated during syncytium development, which suggests that its product has an adverse impact on syncytium or nematode development. The aim of this work was to verify whether *H. schachtii* requires altered *AtMYB59* gene expression to achieve proper developmental conditions for *A. thaliana* roots.

## 2. Results

### 2.1. Expression of AtMYB59 Gene

**Figure 1:**
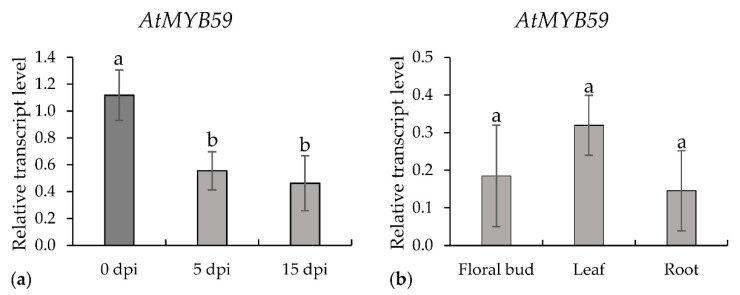
The relative expression levels of *AtMYB59* in syncytia induced by *H. schachtii* in wild-type *A. thaliana*: (**a**) relative transcript levels of *AtMYB59* in roots at 0, 5 and 15 dpi; and (**b**) relative transcript levels of *AtMYB59* in floral buds, leaves and roots of one-month-old plants. The bars show mean values ± standard deviation. a, b homogenous groups. (*p* < 0.05; ANOVA; LSD).

Based on the transcriptome analysis of syncytia induced in *A. thaliana* roots by the beet cyst nematode *H. schachtii* [12], we found that *AtMYB59* was significantly downregulated and presumed that its expression might have a negative impact on the development of syncytia and/or nematodes. Following Szakasits et al. [12], we analyzed the expression level of *AtMYB59* in roots at 5 and 15 days post-infection (dpi), which confirmed the statistically significant downregulation of its expression up to more than 50% of its expression level in wild-type plants at both time points (Figure 1a). Concomitantly, we analyzed *AtMYB59* transcript accumulation in the floral buds, leaves, and roots of one-month-old plants. The levels of *AtMYB59* expression in the flower buds and roots were similar. A slightly higher expression level was found in leaves, but this difference was statistically insignificant compared with flower buds and roots (Figure 1b).

**Figure 2:**
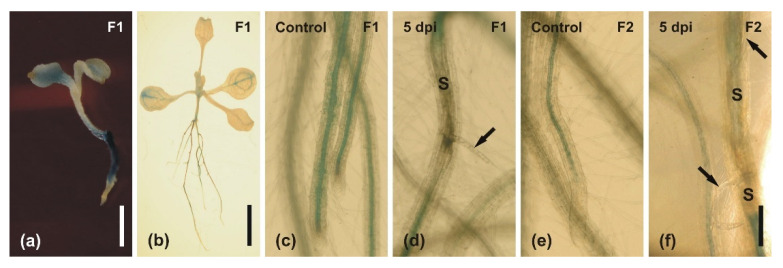
Histochemical staining of GUS activity driven by fragments of *AtMYB59* promoter in transgenic *A. thaliana* plants: (**a**–**c**,**e**) control non-inoculated plants; (**d**,**f**) infected roots with juveniles and syncytia at 5 dpi; (**a**–**d**) GUS activity driven by the F1 (704 bp) fragment of the *AtMYB59* promoter; (**e**,**f**) GUS activity driven by the F2 (507 bp) fragment of the *AtMYB59* promoter; (**a**), three-day-old seedling (slight GUS activity present in cotyledons and strong in roots); (**b**), seven-day-old seedling (GUS activity present in roots and vascular tissues of cotyledons); (**c**) GUS activity under control of the F1 promoter fragment in non-inoculated roots; (**d**) GUS activity under control of the F1 promoter fragment in 5 dpi roots; (**e**) GUS activity under the control of the F2 promoter fragment in non-inoculated roots; (**f**) GUS activity under the control of the F2 promoter fragment in 5 dpi roots. GUS activity was evidenced in the vascular tissues of non-inoculated roots, but it disappeared in nematode feeding sites. Abbreviation: S, syncytium. Arrows indicated juveniles of the nematode. Scale bars: 1 mm (**a**); 10 mm (**b**); 100 µm (**c**–**f**).

GUS activity was investigated in two transgenic lines, where *GUS* expression was controlled by two versions of *AtMYB59* promoter differing in length: F1 is 704 bp and F2 is 507 bp (Figure 2). No differences were observed between these promoter fragments regarding the site and strength of GUS activity in p*Myb59::GUS* homozygotic lines. Both analyzed promoter sequences (F1 and F2) were not activated in the aerial organs (stem, buds, flowers, leaves, or siliques) of flowering four-week-old *A. thaliana* plants. Strong GUS activity was detected in the roots of three-day-old transgenic seedlings of both transgenic lines, and weak GUS activity appeared in the cotyledons (Figure 2a). However, in seven-day-old seedlings, with their first true leaves developed, GUS activity was identified in the vascular tissue of cotyledons and roots (Figure 2b). The promoter::GUS analyses showed that the activity of both promoter versions was mostly restricted to the roots. The GUS activity was detected in the vascular cylinder in the apical parts of the roots, but not in the root meristems or the primordia of the lateral roots in both transgenic lines (Figure 2c,e). The downregulation of GUS activity occurred in regions containing nematode infection sites at 5 dpi (Figure 2d,f) and persisted until 15 dpi in both p*Myb59::GUS* lines (data not shown). Both results, gene expression analysis and GUS activity analysis, confirmed the downregulation of *AtMYB59* expression after nematode infection of *A. thaliana* roots.

### 2.2. Expression of AtMYB59 Gene in Roots or Leaves Treated with Phytohormones

**Figure 3:**
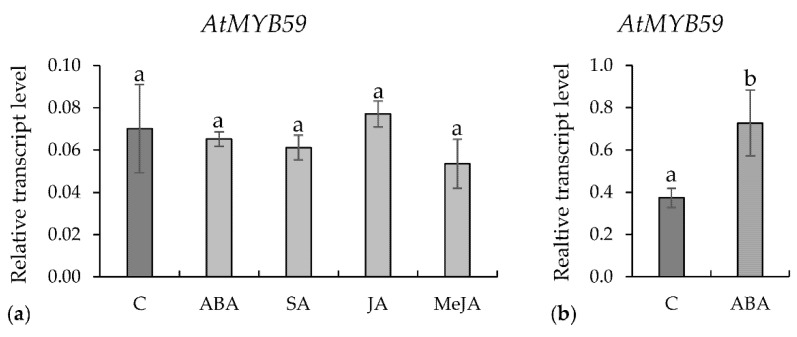
The relative expression level of *AtMYB59* gene in wild-type *A. thaliana* after phytohormones treatment: (**a**) relative transcript levels in roots treated with ABA, SA, JA, or MeJA (100 µM concentration each); and (**b**) relative transcript levels in leaves treated with ABA (100 µM). The bars show mean values ± standard deviation. a, b homogenous groups (*p* < 0.05; ANOVA; LSD).

Expression level of *AtMYB59* was examined in the uninfected roots of the 14-day-old wild-type plants after 24 h of root exposure to jasmonic acid (JA), (-)-methyl jasmonate (MeJA), salicylic acid (SA), or abscisic acid (ABA). There was no statistically significant difference between the *AtMYB59* gene expression level in the control (water-treated) and hormone-treated roots (Figure 3a). In another experiment, the expression of *AtMYB59* was investigated in the leaves of 14-day-old plants 24 h after the foliar application of the aforementioned hormones. *AtMYB59* transcripts accumulation level increased almost two-fold after treatment with ABA (Figure 3b) and this reaction was statistically significant in contrast with the responses to other phytohormones (data not shown).

### 2.3. Infection of Roots with H. schachtii

**Figure 4:**
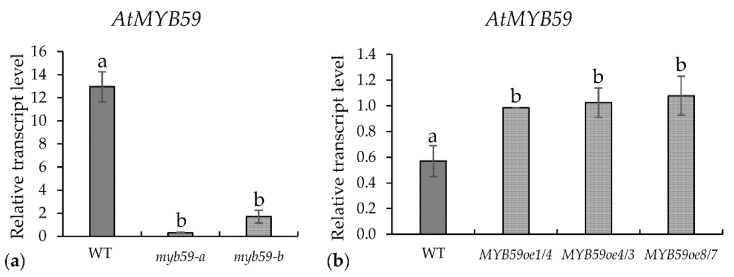
The relative expression levels of the *AtMYB59* gene in roots of wild-type (WT), *myb59-a* and *myb 59-b* mutants, and *MYB59oe* overexpression lines: (**a**) relative transcript levels in *AtMYB59* mutants roots; (**b**) relative transcript levels in *MYB59oe* lines. The bars show mean values ± standard deviation. a, b homogenous groups (*p* < 0.05; ANOVA; LSD).

The potential influence of *AtMYB59* on the *H. schachtii* infection of *A. thaliana* was explored using two T-DNA mutants (*myb59-a* and *myb59-b*), overexpression lines (*MYB59oe1/4*, *MYB59oe4/3* and *MYB59oe8/7*), and Col-0 wild-type plants. The downregulation of *AtMYB59* in *myb59* mutants roots and upregulation in *MYB59oe* lines were confirmed by qRT-PCR (Figure 4). The *MYB59oe* lines used in this work contained full-length cDNA (without both introns) in contrast with the splice variants described by Li et al. [14].

**Figure 5:**
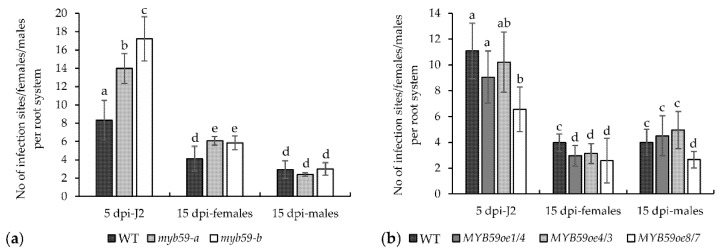
Development of *H. schachtii* juveniles on wild-type, mutant, and *Myb59* overexpressing *A. thaliana* lines at 5 and 15 dpi; (**a**) developmental test comparing the susceptibility of wild-type and *myb59-a* and *myb59-b* mutant plants for infection with beet cyst nematode; (**b**) developmental test comparing the susceptibility of wild-type and different homozygotic *Myb59* overexpressing lines (*MYB59oe1/4, MYB59oe4/3* and *MYB59oe8/7*). The bars show mean values ± standard deviation. a, ab, b, c, d, e homogenous groups (*p* < 0.05; ANOVA; LSD).

In both mutants, a significantly higher average numbers of infecting juveniles per root system at 5 dpi and average numbers of developed females at 15 dpi were observed (Figure 5a). Both values were about 40% higher in mutants than in control wild-type plants. This suggests that the downregulation of *AtMYB59* expression increases the susceptibility of *A. thaliana* to beet cyst nematode. The average numbers of males developed in mutant roots showed no significant change at 15 dpi compared with wild-type plants. In contrast, but in agreement with these results, the average numbers of J2s that invaded the roots of *MYB59oe* lines at 5 dpi were generally lower, but only in the case of the *MYB59oe8/7* line was the difference statistically significant (Figure 5b). The average numbers of females developed in the roots of *MYB59oe* lines at 15 dpi were significantly lower, with differences between 21% and 35% in comparison with the wild-type plants. The average numbers of males developed in the roots of *MYB59oe* lines at 15 dpi were similar to their numbers found on the roots of wild-type plants except for line *MYB59oe8/7*, where the decrease in the number of males was significant. The difference was about 33% in comparison with wild-type plants (Figure 5b). These results show that the upregulation of *AtMYB59* expression leads to the decrease in *A. thaliana* susceptibility to beet cyst nematode. Based on the infection tests results obtained on *myb59* mutants and *MYB59oe* lines, we suggest that *AtMYB59* plays a significant role in the response of *A. thaliana* roots to the parasitism of *H. schachtii* juveniles.

### 2.4. Cellular and Ultrastructural Differences of Syncytia

**Figure 6:**
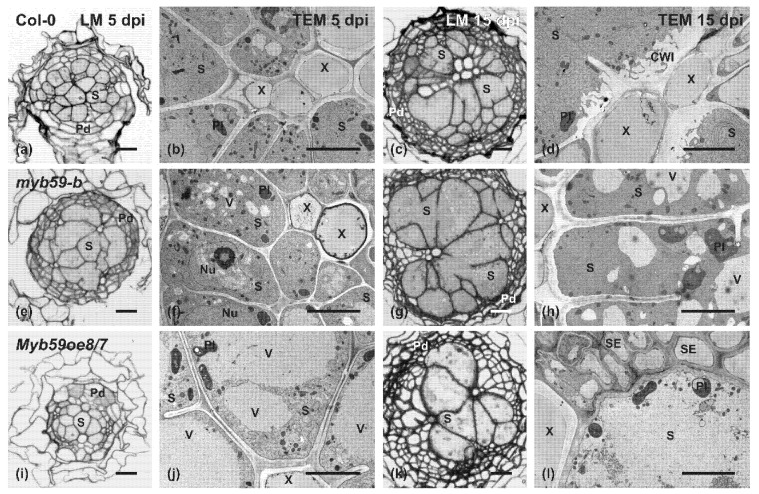
Development of syncytia induced by *H. schachtii* in wild-type, mutant and *AtMYB59* overexpressing genotypes of *A. thaliana*: (**a**,**c**,**e**,**g**,**i**,**k**) light microscopy (LM) images of cross-sections stained with toluidine blue; (**b**,**d**,**f**,**h**,**j**,**l**) transmission electron microscopy (TEM) images; sections were obtained from syncytia induced in the roots of wild-type plants (Col-0) (**a**–**d**), *myb59-b* T-DNA mutant (**e**–**h**), and *AtMYB59* overexpressing line (*MYB59oe8/7*) (**i**–**l**) at 5 (**a**,**b**,**e**,**f**,**i**,**j**) and 15 dpi (**c**,**d**,**g**,**h**,**k**,**l**). Abbreviations: CWI, cell wall ingrowths; Pd, periderm; Pl, plastid; S, syncytium; SE, sieve element; X, xylem; V, vacuole. Scale bars, 20 µm (**a**,**c**,**e**,**g**,**i**,**k**) and 5 µm (**b**,**d**,**f**,**h**,**j**,**l**).

Based on the results of nematode developmental tests and *AtMYB59* expression analyses, the *myb59-b* mutant and *MYB59oe8/7* overexpressing line were selected for detailed investigations of the anatomical and ultrastructural organization of syncytia induced by *H. schachtii* (Figure 6). There were no differences in the anatomy or development of uninfected roots. The roots of mutant and *AtMYB59* overexpressing lines developed typical primary and secondary states of growth as wild-type plants (data not shown).

In all the analyzed genotypes, syncytia were induced and well-developed at 5 dpi (Figure 6a,e,i), and they were only composed of vascular cylinder cells. The syncytia, at their widest region, were surrounded by dividing pericycle cells forming the periderm. In the *MYB59oe8/7* line, the periderm development was the least advanced in comparison with other genotypes at 5 dpi. The surface of the cross-section of the syncytia induced in mutant roots was, at this stage of their development, similar to that of the wild-type plants. In contrast, the syncytia induced in the roots of the *MYB59oe8/7* line were smaller (on cross-sections) than the syncytia induced in the roots of the wild-type or *myb59-b* mutant plants. The difference in their size was mostly a result of the much weaker hypertrophy of syncytial elements. Additionally, fewer cell wall openings were formed between the syncytial elements in the *MYB59oe8/7* line. These anatomical differences became more obvious in 15 dpi syncytia (Figure 6c,g,k). At this developmental stage, on cross-sections obtained from the widest region of the syncytium, the hypertrophy of individual syncytial elements and the size and number of cell wall openings were even higher in the syncytia induced in *myb59-b* mutant roots than in syncytia developed in wild-type plants. Syncytia induced in the roots of *MYB59oe8/7* line were still smaller than those in wild-type plant roots due to the low numbers of cells incorporated into the syncytium and the low numbers of cell wall openings between syncytial elements.

Ultrastructural analysis of syncytia showed similarities between syncytia induced in *myb59-b* mutant and wild-type plants (Figure 6b,d,f,h). These syncytia had an electron-dense cytoplasm with notably smaller vacuoles, enlarged nuclei and nucleoli, and high numbers of plastids, mitochondria and endoplasmic reticulum cisternae. However, in syncytia developed in *myb59-b* mutant roots, small vacuoles were more numerous than in syncytia formed in wild-type plants at both examined time points (Figure 6f,h). Additionally, at 15 dpi in syncytia induced in *myb59-b* roots, numerous huge plastids with a highly developed thylakoid systems were observed (Figure 6h). Similar plastids were not found in syncytia induced in other tested lines. In contrast, syncytia developed in the roots of the *MYB59oe8/7* line showed less electron-dense cytoplasm and lower numbers of endoplasmic reticulum cisternae at 5 dpi than syncytia induced in wild-type roots (Figure 6j vs. Figure 6b). Additionally, the large part of their volume was occupied by large vacuoles or relatively electron-translucent cytoplasm (Figure 6j). Many plastids contained starch grains that were not found in syncytia induced in wild-type plants and *myb59-b* mutants (Figure 6j vs. Figure 6b,f). Only the remains of degraded cytoplasm, plastids, mitochondria, and rarely, the debris of endoplasmic reticulum cisternae were present in syncytia induced in the roots of the *MYB59oe8/7* line at 15 dpi (Figure 6l).

In all examined lines and at both analyzed stages of syncytia development, direct contact was maintained between the outer cell wall of the syncytium and the conductive elements of the vascular cylinder (Figure 6). Additionally, there were no degenerated cells around developing syncytia. In all analyzed genotypes, the cell wall ingrowths systems (similar to that depicted in Figure 6d), in a more or less expanded shape, were formed on the outer syncytial cell wall adjacent to the xylem vessels. Thus, we concluded that the degeneration of syncytia in *MYB59oe8/*7 line was not caused by the limited inflow of water and organic compounds from the conductive elements but was evoked by internal triggers.

### 2.5. cis Regulatory Elements Analysis of AtMYB59 Gene Promoter

Using a New Place bioinformatics tool, we found 57 different classes of *cis* elements (162 in total) in a 704 bp-long promoter sequence of *AtMYB59* gene and 105 (belonging to 42 classes) out of 162 also occurred in the shorter, 507 bp-long, analyzed promoter fragment. Since no difference was discovered in the activity of both promoter fragments in the roots of *A. thaliana* plants before or after infection by *H. schachtii*, the *cis* elements responsible for the downregulation of the *AtMYB59* gene expression in syncytia are likely to remain present in the 507 bp fragment. The bioinformatics analysis indicated 27 *cis* elements that are specific for the 507 bp fragment and absent in the 197 bp fragment by which the 704 bp fragment was reduced. Among these, there were eight ABA- and/or dehydration-responsive *cis* elements, four resistance- and/or wounding-responsive *cis* elements, one auxin- and 1 jasmonate-responsive *cis* element, and 13 other types of *cis* elements that may be related to the response of plants to abiotic or biotic stresses (Table 1).

## 3. Discussion

Transcription factors are regulatory proteins that specifically bind to relevant *cis* acting elements in the promoter region of a gene to activate its expression. The regulation of gene transcription is an important method to modulate plant growth as well as to adjust the plant response to various biotic and abiotic stresses. Transcription factors can be divided into different families according to the specificity of the DNA binding region [15]. *AtMYB59,* analyzed in this work, encodes putative R2R3-MYB protein. The predicted protein sequence contains two nuclear localization signals (NLS) in the R3 repeat, both required for transport to the nucleus [14,16]. The ability of the AtMYB59 protein to regulate transcriptional activity in plant cells was confirmed; it is involved in the regulation of cell-cycle progression and root growth [16].

In previous studies, it was found that *AtMYB59* is differentially regulated in an organ-specific manner as well as after leaves are treated with different phytohormones and subjected to different stresses [14,16]. The transcript accumulation level of *AtMYB59* in leaves, inflorescences, or roots was confirmed in this study; however, transcript synthesis after treating roots or leaves with hormones differed. In this research, we focused on the regulation of *AtMYB59* expression in roots, since they are infested by the plant parasitic nematode *H. schachtii*. The upregulation of *AtMYB59* transcription in roots after hormones treatment did not occur; however, its upregulation in leaves was induced by ABA application. In a previous work [14], the upregulation of *AtMYB59* splice variant 1 transcript synthesis was observed in leaves after JA and SA applications, but not after ABA treatment. Partially similar results were obtained in another study, where plants were treated with a lower concentration of ABA for 6 h [17]. The moderate upregulation of *AtMYB59* expression in leaves and the strong downregulation in roots were also reported [17]. All these results confirm that *AtMYB59* is differentially regulated upon ABA treatment and that the level of its expression depends on the organ, ABA concentration, and treatment duration. The observed differences may have resulted from the three-times-longer exposure time applied in our study (24 h) than in the previous experiment conducted by Li et al. [14], who treated plants for 8 h. Presumably, the plant’s response to the foliar application of hormones is faster when JA and SA are used, whereas the response to ABA, as shown in our work, can appear after prolonged exposure.

ABA is considered a hormone primarily involved in plant responses to abiotic stress, particularly drought and salinity [18]. However, ABA can also play a pivotal role in plant immunity. A set of studies showed that ABA treatment can modulate the plant defense response to pathogens, including parasites. The application of ABA increased the susceptibility of rice and tomato to root-knot nematodes (*Meloidogyne graminicola* or *M*. *javanica*) infection [19,20].

In this work, we identified several ABA- and drought-responsive elements in the fragment of *AtMYB59* promoter. Additionally, *AtMYB59* expression was upregulated after ABA treatment, suggesting that ABA plays a role in the induction of *AtMYB59* expression. However, the regulation of *AtMYB59* gene function by ABA may be liberalized during root infection with parasites, which should be the subject of further investigations.

In opposition to previous research results [14,16,21], where longer *AtMYB59* promoter fragments (2078, 2009, and 1600 bp) were exploited, we analyzed two shorter fragments (507 and 704 bp long) to determine their potential role during root infestation by cyst nematodes. GUS activity driven by 2078 bp-long *AtMYB59* promoter was detected in seedlings, mature leaves, flowers, siliques and stems [14]. Du et al. [21], using a 2009 bp long *AtMYB59* promoter fragment, observed GUS activity in seedling roots (including root hairs). When a 1600 bp-long fragment of *AtMYB59* promoter was used, GUS activity was detected in the hypocotyls and roots (including root hairs), vascular tissue, and root tip meristem, as well in the leaf edges and the pedicels of siliques [16]. In this work, transgenic plants containing truncated versions of *AtMYB59* promoter showed reduced and transient GUS activity in the cotyledons and the vascular cylinder in confined regions of the roots. This result may indicate that *cis* regulatory elements responsible for the activation of *AtMYB59* promoter in other organs were removed by the severe reduction in promoter length. During root infection by juveniles, GUS activity was completely lost early upon invasion at the site of infection and around the developing syncytium. This result shows that *cis* elements responsible for the reduction in *AtMYB59* expression level after nematode attack and during successive nematode feeding and development were preserved in the truncated sequences of *AtMYB59* promoter.

The expression of *AtMYB59* in yeast cells resulted in the inhibition of their proliferation and caused the formation of bi-nuclear cells with high aneuploid DNA content in parallel with the elongation of cell shape [16]. AtMYB59 protein probably affects cell proliferation by interfering with DNA replication, chromosome separation, cell division and cell growth [16]. It was shown that *AtMYB59* transcript accumulation strongly increased in S or S-to-G2 phases of the cell cycle in comparison with the remaining cell cycle phases when its expression levels were low [16]. Our microscopic examinations showed that the root anatomy of mutant and *AtMYB59*-overexpressing plants is the same. However, substantial differences concerning syncytium development and organization clearly appeared. Similar to yeast cells, the overexpression of *AtMYB59* in plants seems to disturb the regular development of syncytia and surrounding secondary cover tissue (periderm). High levels of AtMYB59 protein apparently lead to a lower number of dividing pericyclic cells necessary to properly develop a periderm surrounding the syncytium or cause serious delays in this process. Additionally, syncytia induced in *AtMYB59oe* plants were generally smaller, composed of fewer elements that were interconnected by only a few and small cell wall openings. This may indicate that *AtMYB59* adversely influences syncytium development, not only by interference in the cell cycle and cell division, but also through obvious changes in the properties of the cell walls, which finally results in the development of smaller and less-effective syncytia, which prematurely deteriorate, thus stopping the development of the females of the beet cyst nematode.

Du et al. [21] showed that *AtMYB59* maintains the distribution of K^+^ and the balance of NO_3_¯ between roots and shoots by the positive regulation of *Nitrate Transporter1.5 (NRT1.5)/Nitrate Transporter/Peptide Transporter Family7.3* (*NPF7.3*) transcription in response to low K^+^ stress. It was proven that AtMYB59 protein directly binds to the *NPF7.3* gene promoter. Another study showed that *AtMYB59*, as a negative regulator in calcium (Ca) signaling and homeostasis during Ca deficiency, leads to the regulation of plant growth and stress responses [17].

Considering the aforementioned results and the role of *AtMYB59* indicated in our work in the response of *A. thaliana* to cyst nematode attack, it seems that *AtMYB59* may play a broad role from metabolism modulation to the responses to abiotic and biotic stresses.

Sixteen classes of transcription factors seem to be specific to the plant kingdom [22]. Two of them, MYB and WRKY families, are key players in, inter alia, the plant response to biotic stresses [23,24]. In *A. thaliana*, there are well-evidenced examples of transcription factors that play a role in the plant immune response. Among them are genes such as *AtMYB96*, *AtMYB30*, or *AtMYB46* [25,26,27,28] participating in the responses to viruses, bacteria, or fungi, and against nematodes such as *AtWRKY23* and *AtWRKY72*. The former is probably the best-characterized transcription factor involved in plant–nematode interactions. Its protein was synthesized during the early stages of nematode feeding site development. The knocking down of *AtWRKY23* gene expression resulted in a decreased infection rate of the beet cyst nematode [29]. Another protein, AtWRKY72, is required for full basal defense against *M. incognita* [30]. It is an orthologue of tomato *SlWRKY72a* and *b*, which are transcriptionally upregulated during disease resistance mediated by the *Mi1* nematode resistance gene. It was also shown that 28 out of 66 *WRKY* genes identified in the *A. thaliana* genome were significantly downregulated; only six were upregulated in roots infected with *H. schachtii* [31]. Since they were downregulated in syncytia, *AtWRKY6*, *AtWRKY11*, *AtWRKY17* and *AtWRKY33* genes were studied in detail, and it was confirmed that their silencing is essential for successful nematode development [31]. There is an example available showing that MYB and WRKY transcription factors can influence each other: AtMYB44 regulates defense responses by the transcriptional activation of downstream *AtWRKY70* by direct binding to a conserved *cis* regulatory element in its promoter [32,33].

The role of the *MYB* genes in response to nematode infection is generally poorly understood and examined. It was shown that *AtMYB12* was transiently upregulated in 9 dpi syncytia induced by *H. schachtii*, and the *myb12* mutant was less susceptible to this parasite because the average number of females developed in its roots was lower. It was suggested that *AtMYB12* is more essential for infection and syncytia development than being involved in plant defense. AtMYB12 controls *AtCHS* and *AtFLS1* (encoding chalcone synthase and flavonol synthase, respectively) and they both may contribute to plant defense against nematodes [34]. Recently, the novel *miR858 (microRNA858*)*–AtMYB83* regulatory system in plant–cyst nematode interactions was reported [35]. It was shown that both interactors were transcriptionally upregulated in the syncytia induced by *H. schachtii*. Overexpression of *miR858* led to a reduced susceptibility of *A. thaliana*, whereas the decreased expression of *miR858* enhanced plant susceptibility to *H. schachtii*. Similarly, the overexpression of a non-cleavable coding sequence of *AtMYB83* significantly increased plant susceptibility, whereas *myb83* mutation decreased plant susceptibility [35]. The *miR858*-resistant variant of *AtMYB12* overexpression lines exhibited elevated susceptibility to the nematode. The authors suggested that *AtMYB12* may constitute part of the *miR858/AtMYB83* regulatory loop modulating the plant response to nematode infection [35]. Although transcription factors are proteins that regulate the transcription of other genes, there are also genes encoding transcription factors or microRNAs, which reversely regulate the transcription of primary transcription factors. Thus, the interaction network appears complex and intertwined, and requires further extensive and detailed research.

*AtMYB59* expression is downregulated in infection sites in *A. thaliana* roots during the entire duration of *H. schachtii* parasitism. The inhibition of the activity of the truncated fragments of the *AtMYB59* promoter in infection sites showed that *cis* regulatory elements are located relatively closely to the transcription start site; however, further investigation should be conducted to establish the factors influencing the properties of the regulatory sequences and their activity, as well as the putative transcription regulation of *AtMYB59* by miRNAs.

## 4. Materials and Methods

### 4.1. Plant Material and Culture Conditions

Wild-type ecotype Col-0, T-DNA insertional mutants of *AtMYB59* (*myb59-a* and *myb59-b*), and transgenic lines overexpressing *AtMYB59* (*MYB59oe1/4*, *MYB59oe4/3*, and *MYB59oe8/7*; all in Col-0 genetic background) of *Arabidopsis thaliana* were used in our experiments. Seeds of *myb59-a* (At5g59780; GK-627C09) and *myb59-b* (SALK_137001; with 2 T-DNA insertions, in *AtMYB59 locus* and in the intergenic region between At4g32480 and At4g32490 *loci*) were obtained from the Nottingham Arabidopsis Stock Centre (UK). Their seeds were surface-sterilized in 95% (*v*/*v*) ethanol for 2 min, then in 5% (*v*/*v*) solution of ClNaO (Sigma-Aldrich, St. Louis, MO, USA) for 8 min, and washed three times in excess dH_2_O. Seeds were placed on KNOP [36] or 1/2MS [37] medium in Ø90 mm Petri dishes. They were stratified for 48 h at 4 °C after being placed on the medium. Plants were cultivated under a 12 h day/12 h night photoperiod (125 µmol m^−2^ s^−1^) at 21 °C.

### 4.2. Gene Construct Preparation

Genomic DNA was isolated from frozen *A. thaliana* Col-0 leaves using the Genomic Mini AX Plant kit (A&A Biotechnology, Gdańsk, Poland). Genomic sequences of *AtMYB59* gene promoter were amplified using primers: F1p: CACC/AGCCACTTATCACACACCCA or F2p: CACC/CCTTCTCTCCCTCACACACAC and Rp: GGAACCTATGGCATTCCTCTT. The two obtained DNA fragments covering the promoter sequence had lengths of 704 and 507 bp. The PCR products were cloned into pENTR™/D-TOPO^®^ vector (Thermo Scientific, Waltham, MA, USA) and sequenced. The promoter fragments were subcloned into a pKGWFS7 vector containing the kanamycin resistance gene (*nptII*) and *GFP* and *GUS* reporter genes (https://gatewayvectors.vib.be, accessed on 14 June 2021) using Gateway^®^ LR Clonase^®^ II Enzyme mix (Thermo Scientific).

Uninfected root segments of 14-day-old *A. thaliana* plants were used to amplify the coding *AtMYB59* DNA sequence (splice variant 2). The following primers pair was used: F: CACC/ATGAAACTTGTGCAAGAAGAATACCG and R: CTAAAGGCGACCACTACCATG. The PCR product was cloned into vector pENTR™/D-TOPO^®^ (Thermo Scientific) and sequenced. The coding sequence was subcloned into pK7WG2D vector under the control of *35S* promoter (https://gatewayvectors.vib.be, accessed on 14 June 2021) using Gateway^®^ LR Clonase^®^ II Enzyme mix (Thermo Scientific). The desired gene constructs were transferred into *Agrobacterium tumefaciens EHA105* clone by electroporation (MicroPulser; Bio-Rad, Hercules, CA, USA).

### 4.3. A. thaliana Transformation

Transgenic overexpression lines (*MYB59oe*) with the coding sequence of *AtMYB59* driven by *35S* promoter and transgenic reporter lines with promoter sequences of *AtMYB59* fused with the *GUS* coding sequence (p*Myb59::GUS*) were obtained by the floral dip transformation method [38]. Genotype homozygosity was confirmed using the ratio of kanamycin-resistant to non-resistant T2 plants germinating on kanamycin-containing medium.

### 4.4. Mutants Genotyping

The homozygosity of T-DNA insertion lines was confirmed in the relevant DNA sequence sites using PCR and the site-specific primers for *myb59-a* (LP1: AAGAGGAATGCCATAGGTTCC and RP1: AGTGGGTGGTGATTTTTGATG with o8409: ATATTGACCATCATACTCATTGC (for GABI-KAT)) and *myb59-b*: (LP2: CGCTCTTCTTGTGGAGTCATC and RP2: AAAACAATTCGACCCCTTTTG with SALK_LBb1.3: ATTTTGCCGATTTCGGAAC) under the standard PCR conditions recommended by the SALK collection.

### 4.5. RNA Extraction and RT-PCR

Total RNA was used for cDNA synthesis and semi-quantitative reverse transcription–polymerase chain reaction (RT-PCR) or quantitative real-time-PCR analyses of *AtMYB59* expression. It was extracted from 50 mg of root segments without apical meristems of 14-day-old uninfected *A. thaliana* plants (0 dpi), root segments containing syncytia at 5 or 15 dpi, young leaves, or flower buds. In other experiments, total RNA was also isolated from: (i) uninfected roots treated for 24 h with jasmonic acid (JA), (-)-methyl jasmonate (MeJA), salicylic acid (SA), or abscisic acid (ABA) solutions (Sigma, Saint Louis, MO, USA), each at 100 µM final concentration applied to the roots/culture media; (ii) leaves of 14-day-old *A. thaliana* plants after the foliar application of the aforementioned phytohormones. Total RNA was also extracted from the roots of *myb59-a* and *myb59-b* mutants and *MYB59oe* lines of *A. thaliana* to confirm down- or upregulation of *AtMYB59*.

Collected tissue samples were immediately frozen in liquid nitrogen and homogenized using a Precellys 24 tissue homogenizer (Bertin Technologies, Montigny-le-Bretonneux, France). Total RNA was extracted using a modified method of Chomczynski and Sacchi [39], including a DNase I treatment step in accordance with the manufacturer’s instructions (Thermo Scientific). The concentration and purity of the RNA were validated on a Nanodrop 2000 (Thermo Scientific), and the RNA integrity was checked by electrophoresis in 1% (*w*/*v*) agarose gel.

First-strand cDNA was synthesized from 0.2 μg purified RNA using a RevertAid™ H Minus First Strand cDNA Synthesis Kit (Thermo Scientific) and diluted three times. For semi-qPCR, 1 μL of cDNA preparations was used in each 10 μL reaction with gene-specific primers. The optimal number of PCR cycles was first determined and amplifications were conducted using 27 cycles for the gene expression analysis of infected roots and hormone-treated leaves or 33 cycles for the analysis of leaves, buds and roots. Each cycle consisted of denaturation at 94 °C for 50 s, annealing at 60 °C for 50 s, and elongation at 72 °C for 2 min. An Applied Biosystem 9700 GeneAmp (Applied Biosystems, Foster City, CA, USA) thermal cycler was used. For *AtMYB59* relative gene expression, the following primers were used F: GGAGGCTCCAACGGGAAAAT and R: GTTGGAGAAGCCAGAGGAGG. *Actin2* (AT3g8780) [40] was used as a reference gene and F: CTTGCACCAAGCAGCATGAA and R: CCCCAGCTTTTTAAGCCTTTGATC primers were used.

For the gene expression analysis of roots treated with phytohormones, quantitative PCR analysis was performed using SybrGreen Master Mix (Roche, Basel, Switzerland) and 1 μL cDNA as a template. Reactions were run on a LightCycler 96 (Roche). The obtained results were calculated using tree reference genes: *Actin2* (AT3g18780) F: CTTGCACCAAGCAGCATGAA and R: CCCCAGCTTTTTAAGCCTTTGATC, *Actin8* (At1g49240) F: ATGAAGATTAAGGTCGTGGCA and R: TCCGAGTTTGAAGAGGCTAC, and *GAPDH* (AT1g13440) F: TTGGTGACAACAGGTCAAGCA and R: AAACTTGTCGCTCAATG-CAATC.

### 4.6. Promoter cis Element Analysis

The promoter sequences of *AtMYB59*, 704 and 507 bp long, were analyzed using the online database New Place to localize *cis* regulatory elements [41].

### 4.7. GUS Activity Assay

Histochemical detection of GUS activity was performed as described by Wiśniewska et al. [42]. GUS activity was examined in the leaves, flowers, siliques, seedlings, non-infected roots, as well as the roots containing 5 and 15 dpi syncytia with associated J2s of *H. schachtii*.

### 4.8. Nematode Infection Assay

Cysts of beet cyst nematodes (*Heterodera schachtii* Schmidt) were collected from white mustard (*Sinapis alba* cv. Albatros) roots grown in vitro on KNOP medium. They were incubated in 3 mM ZnCl_2_ (Merck, Darmstadt, Germany) and hatched infective J2s were collected after 6–7 days [36]. J2s were sterilized in 0.05% (*w*/*v*) HgCl_2_ (Poch S.A., Gliwice, Poland) for 2 min, and immediately washed three times in distilled H_2_O. Fourteen-day-old *A. thaliana* plants, wild types, overexpression lines (*MYB59oe*), mutants (*myb59-a* and *-b*), and promoter lines (p*Myb59::GUS*) were grown aseptically and inoculated with 70 J2s per plant under sterile conditions [43]. Inoculated plants were cultivated under the same conditions as described above. The number of infection sites (at 5 dpi) and the number of females and males (at 15 dpi) were counted per plant.

### 4.9. Anatomic and Ultrastructural Analysis

Uninfected roots and root segments containing 5 and 15 dpi syncytia were dissected and processed for microscopic examinations as described by Różańska et al. [44].

### 4.10. Statistical Analysis

The significance of differences in the data was tested using Fisher’s multiple range test and one-way ANOVA. The least significant difference (LSD) was calculated at *p* < 0.05. The RT-PCR experiments were performed in three biological replicates. The nematode infection assay was performed with at least five biological replicates for genotype (n > 30).

## Figures and Tables

**Table 1:** Predicted *cis* elements specific for the 507 bp-long promoter of *AtMYB59*.

Transcription Factor or *cis* Element Name	Signal Sequence	New Place Ref.	Number	Description
ABRELATERD1	ACGTG	S000414	1	ABA- and dehydration responsive
ABRERATCAL	MACGYGB	S000507	1	ABA- and dehydration responsive
ACGTATERD1	ACGT	S000415	2	dehydration responsive
ANAERO1CONSENSUS	AAACAAA	S000477	2	anaerobically induced
**BIHD1OS**	**TGTCA**	**S000498**	**1**	**resistance responsive** *
CATATGGMSAUR	CATATG	S000370	2	auxin responsive
CCAATBOX1	CCAAT	S000030	2	heat shock responsive
CIACADIANLELHC	CAANNNNATC	S000252	1	circadian related
CMSRE1IBSPOA	TGGACGG	S000511	1	carbohydrate metabolite signal responsive element 1
DPBFCOREDCDC3	ACACNNG	S000292	2	ABA responsive
**EBOXBNNAPA**	**CANNTG**	**S000144**	**4**	light responsive, **phenylpropanoid biosynthesis** related
EECCRCAH1	GANTTNC	S000494	1	CO_2_ responsive
GT1CONSENSUS	GRWAAW	S000198	6	light regulated
MYB1AT	WAACCA	S000408	1	Dehydration responsive
MYBCORE	CNGTTR	S000176	2	dehydration responsive, flavonoid biosynthesis related
MYCCONSENSUSAT	CANNTG	S000407	4	dehydration and cold responsive
NODCON1GM	AAAGAT	S000461	1	nodule specific
OSE1ROOTNODULE	AAAGAT	S000467	3	nodule specific
**PALBOXAPC**	**CCGTCC**	**S000137**	**1**	**fungal elicitor, wounding and light responsive**
POLASIG3	AATAAT	S000088	2	polyA signal
**QARBNEXTA**	**AACGTGT**	**S000244**	**1**	**wounding responsive**
RAV1AAT	CAACA	S000314	4	ABA, drought and cold responsive
T/GBOXATPIN2	AACGTG	S000458	1	jasmonate-responsive
TATABOX2	TATAAAT	S000109	1	TATA box
TATABOX4	TATATAA	S000111	2	TATA box
TATAPVTRNALEU	TTTATATA	S000340	1	TATA box-like
WUSATAg	TTAATGG	S000433	1	root meristem specific

* bolded, resistance/wounding-responsive *cis* elements.

## Data Availability

Not applicable.
